# Venous Thromboembolism in Children: From Diagnosis to Management

**DOI:** 10.3390/ijerph17144993

**Published:** 2020-07-11

**Authors:** Giuseppe Lassandro, Viviana Valeria Palmieri, Valentina Palladino, Anna Amoruso, Maria Felicia Faienza, Paola Giordano

**Affiliations:** Department of Biomedical Sciences and Human Oncology, Paediatric Section, University of Bari “A. Moro”, 701024 Bari, Italy; giuseppelassandro@live.com (G.L.); viviana_palmieri@libero.it (V.V.P.); valentinapalladino@hotmail.it (V.P.); amorusoanna@hotmail.it (A.A.); paola.giordano@uniba.it (P.G.)

**Keywords:** thrombosis, paediatrics, risk factors, diagnosis, treatment

## Abstract

Venous thromboembolism (VTE) in children is a rare occurrence, although in recent decades we have seen an increase due to several factors, such as the rise in survival of subjects with chronic conditions, the use of catheters, and the increased sensitivity of diagnostic tools. Besides inherited thrombophilia, acquired conditions such as cardiovascular diseases, infections, chronic disorders, obesity and malignancy are also common risk factors for paediatric VTE. The treatment of paediatric VTE consists of the use of heparins and/or vitamin K antagonists to prevent dissemination, embolization, and secondary VTE. Randomized clinical trials of direct oral anticoagulants in paediatric VTE are ongoing, with the aim to improve the compliance and the care of patients. We reviewed the physiological and pathological mechanisms underlying paediatric thrombosis and updated the current diagnosis and treatment options.

## 1. Introduction

Venous thromboembolism (VTE) in children is a severe issue with high mortality rates and can cause acute and chronic related complications, such as pulmonary embolism, cerebro-vascular events, and post-thrombotic syndrome (PTS) [1,2]. In recent years, the occurrence of VTE in the pediatric population has increased, due to both greater exposure to risk factors, and improved diagnostic techniques [3,4]. The majority of thrombotic events are observed in neonates and adolescents, in relation to the wide range of underlying diseases and interventions. The majority of pediatric subjects with VTE present inherited or acquired risk factors, such as the use of a central venous catheter (CVC), which accounts for more than 90% of neonatal VTE and more than 50% of paediatric VTE [4]. Spontaneous thrombosis in healthy children is rare and represents the biggest challenge regarding treatment, especially in terms of duration.

The treatment of pediatric VTE consists of the use of heparins and/or vitamin K antagonists. Recently, randomized clinical trials of direct oral anticoagulants in paediatric VTE have been reported. These new drugs have the potential to improve care and compliance in children and may represent a new treatment option. In this review, we focus on the pathophysiological mechanisms underlying paediatric thrombosis, the major risk factors, and updating the current diagnosis and new treatment options.

## 2. Pathophysiology and Incidence

VTE occurs when one or more components of Virchow’s triad are activated: changes in blood flow (rheology, stasis), endothelial injury, and hypercoagulability of blood [5]. The thrombosis develops as a result of alterations in the balance between the procoagulant, anticoagulant, and fibrinolytic systems [6]. The incidence of VTE is significantly lower in children compared to adults. During the neonatal period and in infancy, the reduced ability to generate thrombin, the increased ability of macroglobulin alpha 2 to inhibit thrombin, and the antithrombotic potential of the vessel wall, play a protective role in the development of thrombosis [7]. Furthermore, the children are not exposed to vascular endothelium damage due to hypertension, diabetes, hypercholesterolemia or acquired thrombotic factors, such as smoking or antiphospholipid antibodies [7]. On the other hand, the presence of chronic diseases, such as obesity and diabetes, predispose endothelial dysfunction and cardiovascular pathologies early in childhood [8,9,10]. 

The annual incidence of VTE has been estimated to be 0.07–0.14 per 10,000 healthy children, and 5.3 per 10,000 paediatric hospital admissions [5,6], with a higher occurrence in neonates (0.51/10,000) and in neonatal intensive care units (0.24/10,000) than in early to mid-childhood, and a second peak during adolescence [4]. Despite the low incidence, VTE is increasingly observed and diagnosed in paediatric patients, due to the greater understanding of this disease, the increased survival rate in patients with chronic conditions, and the more frequent use of catheters and interventional procedures [11]. The natural history of paediatric VTE remains unclear. The recurrence risk is reported to be between 10–15% [12]. The incidence of PTS varies from 10% to 60% in relation to the tools used for its assessment. Currently, there is no consensus about the clinical implications of PTS in children [13,14,15]. The reported VTE mortality rate from registry ranges from 0% to 3.7% [16].

## 3. Ethnicity

The incidence of recurrent VTE varies depending on gender, the type of thromboembolic event and race. Although there is evidence that the prevalence of VTE varies significantly among different ethnic groups, the genetic and/or clinical basis for these differences remain unknown. African-American patients have a significantly higher rate of VTE, particularly following exposure to acquired risk factor, such as surgery, medical illness or trauma, and display more pulmonary embolisms than deep-vein thromboses compared to Caucasian and other racial groups. Asians/Pacific Islanders have a 70% lower prevalence of idiopathic and provoked VTE. Hispanics have a significantly lower prevalence of VTE compared to Caucasians, but higher than Asians/Pacific Islanders. Ethnicity should be considered as an important factor in the risk-stratification of patients with suspected VTE or patients at some risk of developing VTE [17].

## 4. Risk Factors

Idiopathic VTE is not common among children as it usually starts as a complication of a primary disease or after interventions [18]. Inherited risk factors should be considered in both provoked and unprovoked thromboses in children [18]. Usually, acquired factors including use of a CVC, malignant diseases, obesity, infections, cardiopathy, and nephrotic syndrome can be found in the majority of cases of paediatric thrombosis [19,20,21]. Use of a CVC is the most common cause in newborns [22,23,24,25]. The occurrence of catheter-related thrombosis in newborns does not require the testing of hereditary thrombophilia [12].

### 4.1. Inherited Risk Factors

Genetic factors play an important role in the development of paediatric VTE [26]. Inherited thrombophilia is a coagulation disorder associated with an increased predisposition to develop thrombosis [27]. In the presence of a clear family history of thrombophilia (early stroke and myocardial infarction <45 years, thrombophlebitis episodes or pulmonary emboli after delivery), thrombophilia testing for hereditary disorders should be considered [12]. Diagnostic thrombophilia testing is also recommended for neonates, children and adolescents with unprovoked and recurrent VTE, in particular when no triggering factor is present [28]. The most common inherited thrombophilic defects include protein S (PS) deficiency, protein C (PC) deficiency, anti-thrombin (AT) deficiency, factor V Leiden (FVL) G1691A polymorphism, and prothrombin (FII) G20210A polymorphism [26,27,28]. The inherited conditions which may determine serious thrombotic risks in children are the homozygous deficiency of AT and the homozygous or combined heterozygous of PC, PS or AT deficiency [29], while the FVL or FII carrier state represent a case of ‘low-risk thrombophilia’ [12,27]. PC and PS are vitamin K-dependent natural anticoagulants which act together to deactivate coagulation cofactors V and VIII [27,30]. PC and PS deficiency can occur in homozygous, heterozygous, or compound heterozygous form, and a severe deficiency of these proteins has been linked with neonatal purpura fulminans [31,32]. AT inhibits thrombin and activated factors IX, X, and XI, and its activity increases in the presence of heparin and endogenous heparin-like substances [33]. AT deficiency is one of the rarest forms of congenital thrombophilia, occurring as an autosomal dominant disorder [27]. The FVL and FII mutations are the most common genetic thrombophilic defects which predispose an individual to VTE [34]. The FVL mutation consists of the substitution of glutamine by arginine at position 506 causing FV resistance to the anticoagulant action of activated PC, with an excess of FVL resulting in a hypercoagulable state [34]. FII is characterized by a point mutation (G20210A) in the 3′ untranslated region (UTR) of the prothrombin gene [27]. This mutation causes increased levels of prothrombin that are associated with a greater VTE risk, probably due to a resistance to activated PC, secondary to hyperprotrombinemia [35]. Testing for methylenetetrahydrofolate reductase (MTHFR) mutations and homocysteine levels should no longer be included in thrombophilia panels [36,37].

### 4.2. Acquired Risk Factors

Use of a CVC is the main acquired risk factor associated with VTE, with an incidence rate of more than 90% in newborns and more than 50% in children [18,19]. The incidence of catheter-related thrombosis ranges between 1% and 70% [38,39]. CVC use can lead to different forms of thrombosis, including the formation of clots at the tip of the catheter (ball-valve clot), fibrin sheaths around the catheter and thrombi in the catheterized vessel [19,40]. Patients with CVC-associated thrombosis may develop other illnesses such as catheter occlusion, loss of venous access, embolism, infection, and PTS [40].

Infections or bacterial toxins can lead to uncontrolled inflammation that alters endothelial, epithelial, immune, and mitochondrial cell homeostasis, resulting in multiple organ system failures. Altered coagulation with bleeding and thrombosis, and immune dysregulation with immune depression and macrophage activation, are associated with multiple organ dysfunction phenotypes that are related to environmental exposure and host genetics. 

Specific therapies include: eculizumab and/or plasma exchange for thrombocytopenia; intravenous immunoglobulin (IVIG) and/or rituximab for lymphoproliferative activity; and IVIG, methylprednisone, and/or anti-inflammatory biologics for macrophage activation syndrome [41].

Paediatric oncologic patients are at high risk of VTE due to disease and treatment-related factors, e.g., chemotherapy (asparaginase, steroids), CVC use and surgery [42]. The occurrence of VTE depends on the type of cancer, and acute lymphoblastic leukemia (ALL) is the most common malignancy associated with thrombosis in children (3–14%) [43,44,45,46]. Different factors may contribute to the thrombosis risk in ALL children and in particular, the thrombin activation which has been observed at the time of the diagnosis as well as after several months of therapy [43]. 

Congenital heart disease (CHD) represents the main risk factor for VTE in children. The hepatic hypo perfusion, as a result of the impaired heart function, causes anticoagulant deficiencies. Furthermore, many patients with CHD undergo open-heart surgery and require placement of a CVC [47]. In addition, it has been proven that vascular anomalies and malformations, in particular venous malformations (VMs), can increase the risk of developing thrombosis [48]. 

The nephrotic syndrome is predisposed to thrombosis, due to AT and PS deficiency, with a rate of 9–36% [49,50]. VTE has also been observed in thalassemia major patients with a prevalence ranging from 2.5% to 4%. The hypercoagulable state in these patients has been attributed to a variety of hemostatic alterations, including platelet hyperaggregability, PC and AT deficiency, and procoagulant alterations of red cells [51].

Over the past two decades VTE frequency has increased in adolescents due not only to the advancement of diagnostics, but also an increase in chronic conditions, obesity and oral contraceptive use. 

Obesity increases the risk of VTE through several mechanisms including, endothelial dysfunction, amplified activity of the coagulation cascade, inhibition of the fibrinolytic process, and oxidative stress [52,53,54,55]. In obese children, a sedentary lifestyle and prolonged video console usage may be additional factors leading to VTE [56].

## 5. Diagnosis

Diagnosis is based on clinical examination and instrumental investigations. Clinical manifestations depend on the affected blood vessel, the degree of vessel occlusion and the situation of the affected organ. The major clinical manifestations are growth impairment of the involved limb or loss of organ function. Acute onset of swelling and pain represent the most frequent clinical manifestations of VTE affecting a limb (Table 1). Measurement of the circumference of the limb and some specific clinical investigations may help in the diagnosis of suspected lower limb VTE in children. These include calf pain felt in either dorsiflexion of the foot (Homan’s sign), calf compression (May’s sign), or sole pain caused by its compression (Payr’s sign).

The diagnosis of a thrombophilic state should be based on at least two laboratory results. Repeating the tests 3–6 months after the initial diagnosis of acute thrombosis, when an anticoagulant is not used, should be considered. Specific diagnostic laboratory tests to confirm diagnosis of thrombosis are not available. High D-dimer values cannot be used for diagnosis of paediatric VTE, as there are no clinical studies confirming their effectiveness in the paediatric population [39]. Doppler and ultrasonography (US), echocardiography, computed tomography (CT) and magnetic resonance imaging (MRI) are normally used to confirm clinical diagnosis. Compression Doppler ultrasonography is the preferred method of instrumental investigation for the diagnosis of thrombosis in children. Venography is considered the gold standard for diagnosis of deep venous thrombosis, but in children it is not used frequently.

## 6. Treatment

The treatment of paediatric VTE involves the use of heparins [unfractionated heparin (UFH), low-molecular-weight heparin (LMWH)], and/or vitamin K antagonists (VKAs)]. Attack therapy should be performed with heparin for the first 7–10 days, followed later by overlapping it with LMWH or VKAs [39,57,58].

The 2012 American College of Chest Physicians guidelines for the treatment of paediatric VTE suggest an anticoagulant duration of 3 months for provoked VTE, 6–12 months for a first unprovoked VTE, and an indefinite period for recurrent unprovoked VTE [29]. Prolonging the prophylaxis time should be considered for children with a high risk of hereditary thrombophilia [12,59]. UFH may be used for emergency events or during surgical procedures, as it is neutralized by protamine. Regarding the disadvantages, activated partial thromboplastin time (aPTT) must be monitored several times a day. Moreover, the risk of heparin induced thrombocytopenia (HIT) and of osteoporosis should also be considered [60,61]. Doses of 75 to 100 U/kg are recommended to maintain therapeutic APTT values at 4 to 6 h post-bolus. Maintenance UFH doses are age dependent: infants need the highest doses (on average 28 U/kg per hour) and children need slightly lower doses (on average 20 U/kg per hour). The doses of UFH required for older children are similar to the weight-adjusted requirements in adults (18 U/kg per hour) [1]. LMWH is the first-choice drug in paediatric VTE because it can be administered subcutaneously and it requires no monitoring of Prothrombin time (PT) and aPTT [39,62,63]. Testing for anti-FXa activity in relation to dosage should be carried out at least 4–6 h after administration. The therapeutic range is 0.5–1.2 IU/mL, whereas the prophylactic range is 0.2–0.4 IU/mL [29,39]. In neonates, the mean dose is 1.62–2 mg/kg twice daily, whereas in infants, the mean dose ranges from 1.12 to 1.9 mg/kg twice daily. The main advantages include a lower risk of HIT and osteoporosis [59]. VKAs are used if there is any unprovoked VTE or significant chronic risk factors for recurrent VTE [64]. Bleeding is the main complication of VKA therapy [58]. Warfarin is the most commonly used VKA. 

The therapeutic range of INR (International Normalized Ratio) is 2.0–3.0. Warfarin usually starts at 0.1 to 0.2 mg/kg (Table 2) [58]. VKAs are difficult to manage in children due to their tablet-only formulation, diet, the need for frequent laboratory monitoring, and the higher doses required in neonates [63,65]. The monitoring should consider intercurrent infections, other medication changes, and diet [59]. 

In the literature, few indications for thrombolytic agents have been reported as they cause an increased risk of cerebral hemorrhages [66]. Therefore, using thrombolytic drugs is only recommended in the case of widespread thromboses, within 2 weeks of the onset of symptoms, with organ distress, or in a case of massive pulmonary embolism [67]. 

Presently, several direct oral anticoagulants (DOACs) are undergoing evaluation in paediatric development programs [68,69,70,71,72,73]. Randomized clinical trials for DOACs in paediatric VTE are ongoing. DOACs recently approved by the FDA, such as dabigatran, rivaroxaban, apixaban and edoxaban, appear to offer potential advantages over VKAs and LMWH. However, key questions have arisen regarding their potential off-label clinical application in paediatric thromboembolic disease [69]. Rivaroxaban is an oral, direct inhibitor of activated factor X (factor Xa), used for the treatment of VTE in adults and is administered as a fixed dose. Male et al. reported that in children with various manifestations of VTE, rivaroxaban treatment with a weight-adjusted dose, body targeting the therapeutic exposure range of young adults, results in a low risk of recurrent VTE and clinically relevant bleeding, similar to standard therapy [72]. Moreover, treatment with rivaroxaban results in a greater reduction in thrombus mass compared to standard therapy. However, further studies on the effectiveness and safety of rivaroxaban are needed. Additionally, the pharmacokinetic and pharmacodynamic profile of the established rivaroxaban regimens need to be confirmed for a longer treatment duration [73]. 

In conclusion, the availability of rivaroxaban as an oral suspension will eliminate the need for dosage adjustments of adult formulations and will substantially reduce the number of injections required for standard anticoagulant treatment and consequent blood sampling. The paediatric rivaroxaban regimen will represent a beneficial alternative treatment for children in the future. 

## 7. Conclusions

Paediatric thrombosis is increasingly recognized and diagnosed. In most children, it results from underlying conditions such as infections, cancer, cardiopathy, chronic inflammatory diseases, or the use of CVCs. Unprovoked paediatric thrombosis is rare, and in this instance, it is advisable to perform thrombophilia tests. Diagnostic thrombophilia testing should also be performed in the case of recurrent thrombosis and when there is a clear family history of thrombophilia. Personal and family history and a detailed clinical examination are important in the diagnosis of thrombosis. Instrumental investigations, supported by laboratory ones, can help to confirm diagnosis. Treatment of childhood thrombosis is based on the administration of heparins and/or vitamin K antagonists. The development of new drugs such as direct oral anticoagulants will improve compliance with therapy in children, while maintaining the same level of effectiveness.

## Figures and Tables

**Table 1:** Common symptoms of venous thrombosis modified by [39].

SITE OF THROMBOSIS	SYMPTOMS
Limb	Pain, edema
Superior vena cava	Headache, neck pain, edema of the neck and head
Inferior vena cava	Tenderness and edema in the lower limbs, abdominal pain
Splenic vein	Abdominal pain in the left upper quadrant, splenomegaly
Portal vein	Abdominal pain, splenomegaly
Renal vein	Flank pain, hematuria
Hepatic vein	Pain in the right upper quadrant, hepatomegaly
Mesenteric vein	Abdominal pain
Pulmonary embolism	Chest pain, cough, respiratory failure, dyspnea
Cerebral sinuses	Headache, vomiting, focal neurologic signs, lethargy, asthenia

**Table 2:** Adjustment of VKA dose according to INR modified by [58].

DAYS OF THERAPY	INR	ACTION
**DAY 1**	1–1.3	Give 0.1–0.2 mg/kg orally (maximum 5 mg)
**DAYS 2–4**	1.1–1.3 1.4–1.9 2.0–3.0 3.1–3.5 >3.5	Repeat initial dose 50% of initial dose 50% of initial dose 25% of initial dose Stop therapy, check INR; when INR <3.5 restart at 50 % of initial dose
**OTHER DAYS**	1.1–1.4 1.5–1.9 2.0–3.0 3.1–3.5 >3.5	Increase dose (+20%) Increase dose (+10%) Continue same dosage Decrease dose (−10%) Stop therapy, check INR; when INR <3.5 restart at 20 % of dose
